# Pneumococcal Meningitis Complicated by Subarachnoid Hemorrhage and Tonsillar Herniation

**DOI:** 10.7759/cureus.9994

**Published:** 2020-08-24

**Authors:** Vrinda Vyas, Gowthami Kanagalingam, Zaid Siddique

**Affiliations:** 1 Internal Medicine, State University of New York Upstate Medical University, Syracuse, USA; 2 Radiology, State University of New York Upstate Medical University, Syracuse, USA

**Keywords:** bacterial meningitis, subarachnoid hemorrhage, tonsillar herniation, cerebral edema, complications of meningitis

## Abstract

Bacterial meningitis is a fatal infectious disease with an annual incidence of four to six cases per 100,000 adults. The most common pathogens associated with this condition are *Streptococcus pneumoniae, Neisseria meningitides, and Hemophilus influenzae*. Mortality rates range between 10 and 40% despite the availability of highly effective antibiotic therapy, and severe neurological damage affects 30-52% of survivors. The causes of death in patients with pneumococcal meningitis are multifactorial and involve both neurological complications such as cerebral edema, hydrocephalus, infarction, and septic sinus or venous thrombosis and systemic complications such as septic shock, disseminated intravascular coagulation, and acute respiratory distress syndrome. We present an unfortunate case of a 42-year-old woman with asplenia and sickle cell disease, admitted for pneumococcal meningitis, who developed diffuse cerebral edema leading to tonsillar herniation and aneurysmal subarachnoid hemorrhage (SAH) with a fatal outcome. To the best of our knowledge, this is the only case ever reported of meningitis complicated by both SAH and brain herniation.

## Introduction

Brain herniation with the consequent cessation of cerebral circulation is the most important cause of death in meningitis patients and occurs due to increased intracranial pressure (ICP). In this report, we present the case of a patient who had findings suggestive of diffuse cerebral edema on imaging, which ultimately proved fatal due to tonsillar herniation in conjunction with aneurysmal rupture and subarachnoid hemorrhage (SAH). We present this case to highlight the rare but fatal consequences of severe meningitis even with appropriate and timely treatment.

## Case presentation

**Figure 1:**
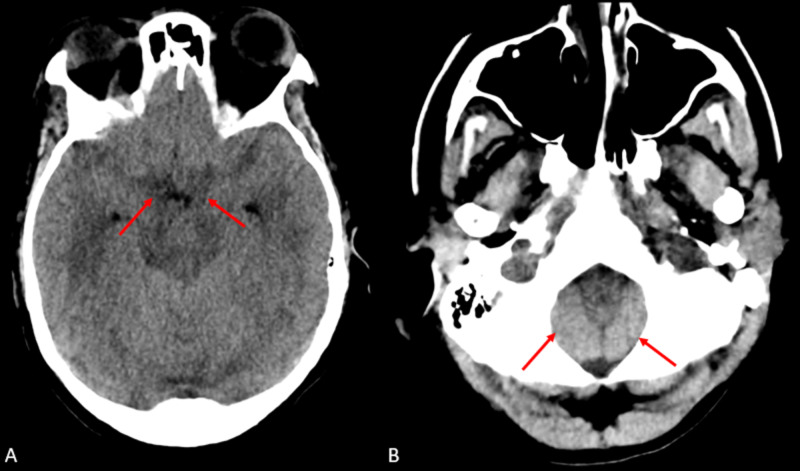
Axial CT head without contrast on presentation (A) There is intermediate density within the suprasellar cistern which could be secondary to exudates from meningitis (red arrows). (B) There is crowding of the foramen magnum with low-lying cerebellar tonsils (red arrow) CT: computed tomography

A 42-year-old woman with a history of sickle cell disease and asplenia presented with a five-day history of left-sided ear pain, headache, and altered mentation. Physical exam revealed nuchal rigidity and photophobia. She had leukocytosis of 25.9 x 10­­­­3/uL and lactate of 6.3 mmol/l. The chest X-ray showed a right middle lobe infiltrate. CT scan of the head showed cisternal effacement without mass lesion and low-lying cerebellar tonsils (Figure 1).

The patient was admitted to the medical intensive care unit (ICU). Neurosurgery was consulted, and they did not recommend any acute intervention for the CT head findings. Lumbar puncture (LP) was performed; cerebrospinal fluid (CSF) analysis was consistent with bacterial meningitis as noted in Table 1.

**Table 1:** Cerebrospinal fluid analysis on presentation CSF: cerebrospinal fluid

Parameters	Reference range	Patient sample
Red blood cell count	<2/uL	124 (H)
Total nucleated cells	<5/uL	2,967 (H)
Color, CSF	Clear	Yellow
Glucose, CSF	40–70 mg/dL	<3 (L)
Protein, CSF	15–45 mg/dl	1,878 (H)

The patient was started on ceftriaxone, vancomycin, ampicillin, and dexamethasone. Hematology was consulted for possible sickle cell crisis, and they recommended intravenous hydration and adequate pain management. ENT was consulted for the evaluation and management of purulent otitis media. A left ear tympanostomy tube was placed. The patient was intubated the next day for worsening mentation. CSF cultures eventually grew *Streptococcus pneumoniae.*

**Figure 2:**
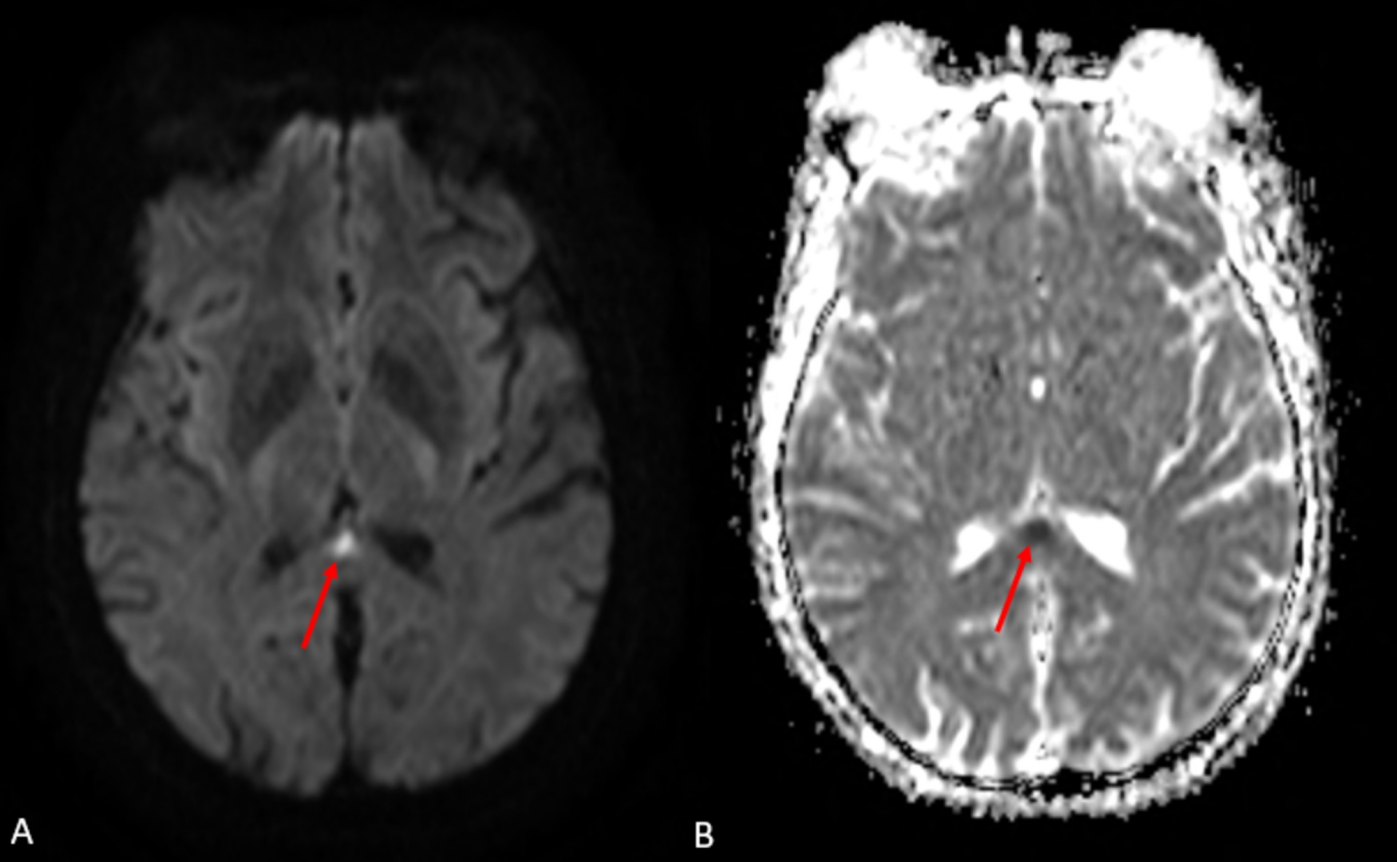
Axial MRI with (A) diffusion-weighted imaging (DWI) sequence and (B) apparent diffusion coefficient (ADC) sequence The images show increased signal within the splenium of the corpus callosum on the DWI (A) with corresponding signal dropout on the ADC (B) (red arrow). This finding may be secondary to metabolic derangement, focal infarct, or infection MRI: magnetic resonance imaging

Sedation was weaned off four days after intubation, and the patient was noted to have right upper extremity weakness, MRI brain showed restricted diffusion in the splenium of the corpus callosum, which was thought to be either secondary to ischemic stroke given her history of sickle cell disease or from inflammation related to meningitis (Figure 2).

**Figure 3:**
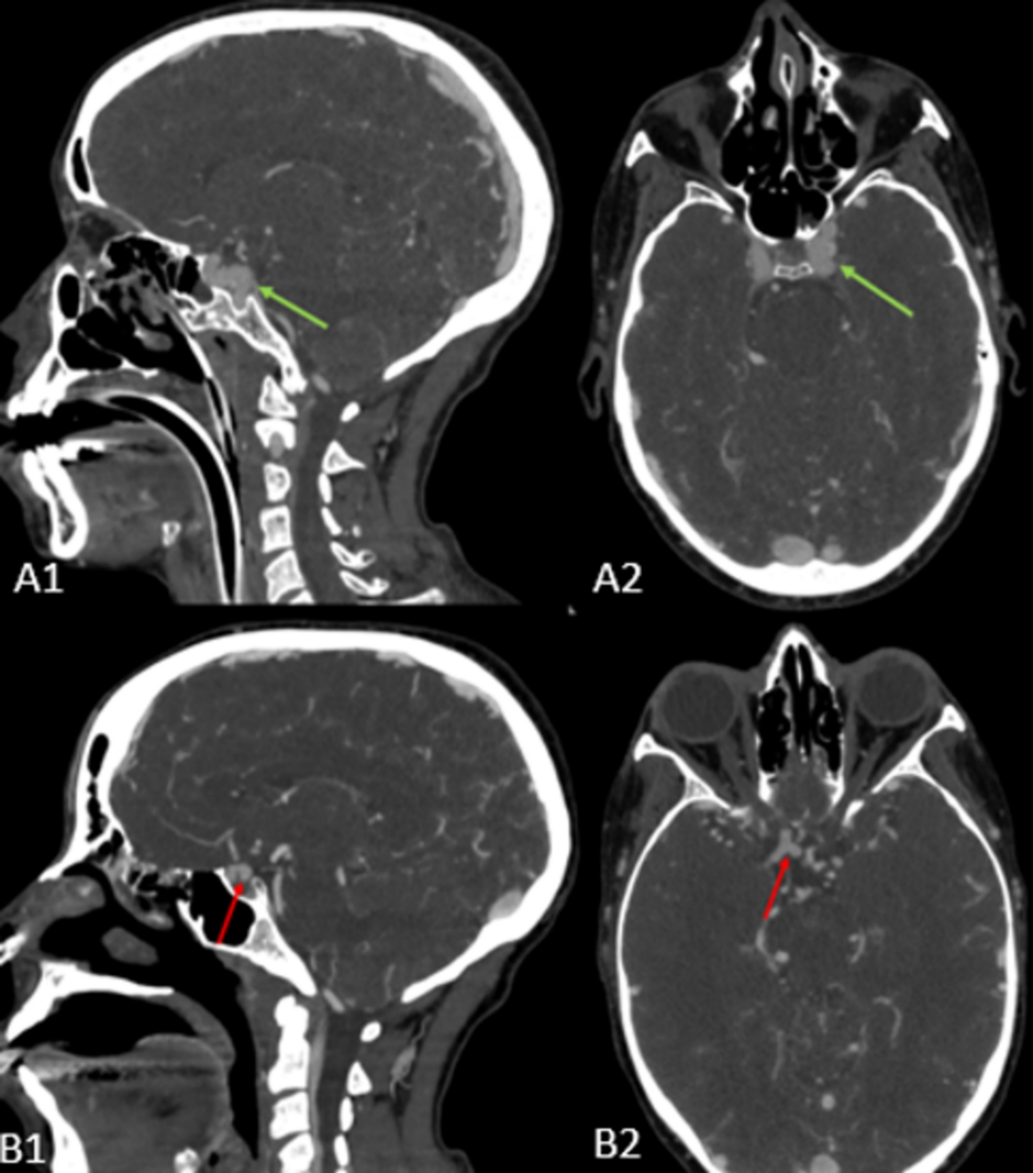
CTA in the coronal (A1, B1) and axial (A2, B2) planes Figure A shows fusiform aneurysm of the cavernous segment of the left internal carotid artery (green arrow). Figure B shows saccular aneurysm of the supraclinoid segment of the right internal carotid artery (red arrow) CTA: computed tomography angiography

At that time, neurology was consulted, and they recommended repeating LP and CT angiography (CTA) head and neck. CTA showed 8-mm fusiform aneurysm in the left cavernous internal carotid artery (ICA) and a 4.4-mm saccular aneurysm in the right supraclinoid ICA (Figure 3).

**Figure 4:**
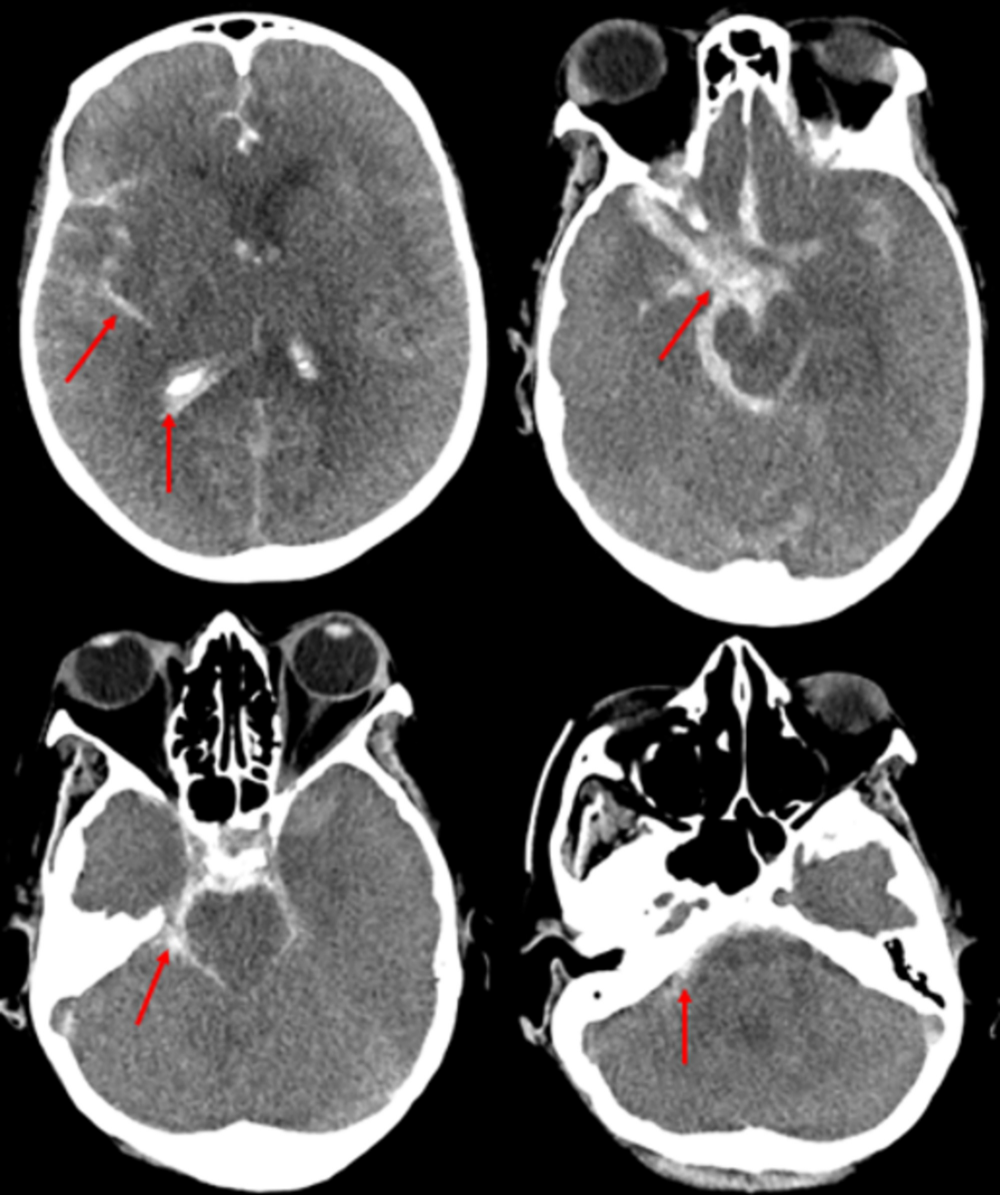
Axial CT head without intravenous contrast There is a large subarachnoid hemorrhage extending into the sulci, ventricles, and basal cisterns (red arrow). There is diffuse cerebral edema with effacement of the sulci and loss of the gray-white differentiation CT: computed tomography

The patient showed persistent clinical improvement and was extubated the next day; half an hour post-extubation, the patient became hypotensive, flaccid, and unresponsive. She was then re-intubated emergently. On examination, the patient was comatose with bilateral pupils being 4-mm dilated and non-reactive, with no cough or gag reflex. Emergent repeat CT head and neck showed a diffuse SAH with cerebellar tonsillar herniation with hypodense brainstem and an absence of intracranial blood flow (Figure 4). The patient was transferred to the neuroscience ICU emergently.

Neurosurgical intervention was not expected to alter outcomes at that time. Brain death testing was performed, and the patient was responsive to noxious stimuli, excluding the diagnosis of brain death. Obstructive hydrocephalus was noted on repeat CT head the following day. Neuroprotective measures were instituted, but there was no improvement in the patient’s clinical state; hence, the patient's family decided to withdraw care and institute comfort measures only, following which she was extubated per family's wishes and passed away the same day.

## Discussion

Acute bacterial meningitis is a medical emergency and results in substantial morbidity and mortality despite the availability of effective antimicrobial therapy [1,2]. Pneumococcal meningitis can cause both neurological (cerebral edema, hydrocephalus, infarction, and septic sinus or venous thrombosis) and systemic complications (septic shock, disseminated intravascular coagulation, and acute respiratory distress syndrome) [3].

Cerebrovascular complications such as thrombosis, vasculitis, acute cerebral hemorrhage, and aneurysm formation are potential complications of bacterial meningitis but are relatively rare [4]. A few other rarer cerebrovascular complications have been described in isolated case reports including hemorrhagic stroke, thrombotic stroke, and SAH due to meningitis. Our patient was noted to have an 8-mm fusiform aneurysm in the left cavernous portion of ICA and a 4.4-mm saccular aneurysm in the right supraclinoid ICA on the CTA obtained a day prior to her catastrophic SAH occurrence.

Cerebral edema, increased intracranial blood volume, and disturbances in CSF circulation are the main factors leading to increased ICP in bacterial meningitis. Intracranial hypertension, especially when combined with signs of imminent brainstem compression, reflects higher mortality than in patients with uncomplicated bacterial meningitis [5]. Pneumococcal meningitis can be associated with cerebral edema, in as many as 5.7%-29% of patients with cerebrovascular complications [6]. Cerebral edema in acute bacterial meningitis is caused by the inﬂammatory reaction due to the release of toxic mediators as a result of bacterial lysis, and the subsequent disruption of the blood-brain barrier causing disturbances of normal hemostasis mechanisms of brain volume regulation [6].

About 2% of patients with meningitis can develop quadriplegia as a consequence of tonsillar herniation, myelitis, cord infarction, arachnoiditis, and epidural abscess formation [6]. Our patient, unfortunately, had a cerebellar tonsillar herniation with a consequent decrease in intracranial perfusion. Case reports have shown favorable recovery with aggressive steroid use and ICP-decreasing therapy in patients with tonsillar herniation in pneumococcal meningitis [6]. However, in our patient, there was no improvement despite timely steroid use.

It is important to note that our patient had an isolated right upper extremity weakness associated with restricted diffusion in the splenium of the corpus callosum. Of note, infarcts of the corpus callosum are rare because of its rich vascular supply. Sometimes, imaging findings suggestive of acute stroke can in fact be related to other conditions that mimic a stroke radiologically, such as an infectious process like brain abscess, neoplasm, acute demyelinating plaque, encephalitis, epilepsy, and hypoglycemia. There have been case reports of reversible restricted diffusion of the splenium of the corpus callosum in viral meningitis but none in bacterial meningitis [7].

To our knowledge, this is the first reported case of an adult with sickle cell disease who developed pneumococcal meningitis, which was complicated by tonsillar herniation with concomitant aneurysmal rupture and SAH.

## Conclusions

Over the past decades, the incidence of acute bacterial meningitis has decreased, largely due to vaccinations against* Streptococcus pneumoniae*, *Hemophilus influenzae,* and *Neisseria meningitides,* but there still remains a significant burden of disease in children and the elderly. Even with appropriate antimicrobial therapy, mortality remains high, and adjunctive therapies with corticosteroids are beneficial only in certain circumstances. While intracranial hypertension and cerebral herniation are well-recognized consequences of bacterial meningitis, our patient developed concomitant SAH from aneurysmal rupture due to change in ICP. This case report highlights the catastrophic consequences of bacterial meningitis despite appropriate treatment. We aim to sensitize physicians to the fatal neurological sequelae of pneumococcal meningitis.
